# Early Osteoporosis Risks and Associated Factors among Caregivers Working in Disability Institutions: IOF One-Minute Osteoporosis Risk Check

**DOI:** 10.3390/ijerph17093319

**Published:** 2020-05-10

**Authors:** Lan-Ping Lin, Wei-Ju Lai, Shang-Wei Hsu, Jin-Ding Lin

**Affiliations:** 1 Department of Senior Citizen Care and Welfare, Ching Kuo Institute of Management and Health, Keelung 203, Taiwan; lanping518@gmail.com; 2 School of Public Health, National Defense Medical Center, Taipei 144, Taiwan; weiju.lai1990@gmail.com; 3 Department of Healthcare Administration, Asia University, Taichung 354, Taiwan; shangweihsu@gmail.com; 4 Institute of Long-Term Care, Mackay Medical College, New Taipei City 252, Taiwan

**Keywords:** osteoporosis, bone fracture, One-Minute Osteoporosis Risk Test, health promotion, exercise

## Abstract

This study employed the International Osteoporosis Foundation’s One-Minute Osteoporosis Risk Test to examine factors related to the osteoporosis risk of institutional caregivers. In this cross-sectional study, a self-developed structured questionnaire comprising the One-Minute Osteoporosis Risk Test was used to obtain data on the caregivers’ demographic data, health habits, working style, and osteoporosis risk. Seven disability welfare institutions were selected as research sites, and 465 copies of questionnaires were distributed to the institutions’ employees, with 455 valid responses collected for a valid return rate of 98%. SPSS for Windows (Version 20.0) was used to analyze questionnaire data; descriptive-statistical frequency, a χ^2^ test, and logistic regression were used to determine the correlation between demographic data, health habits, working style, and osteoporosis risk. The results revealed that primary risk factors include <30 min of daily exercise (38%), lack of dairy product or calcium tablet intake (28%), and <10 min of daily outdoor activity or not taking vitamin D supplements (29.9%). In total, 395 (86.8%) of the respondents scored less than 5 in the osteoporosis risk test; the remaining 60 (13.2%) scored 5 or higher, revealing a high risk of early osteoporosis. An independent variable analysis revealed that the risk factors of early osteoporosis include age, education level, having undergone bone density tests, prior disease diagnosis, long-term medication use, physical fitness, dietary habits, and average time of exposure to sunlight. In the multivariate analysis, poor physical fitness (odds ratio [OR] = 2.18, 95% confidence interval [CI]: 1.12–4.27, *p* = 0.023) and average daily time of exposure to sunlight (OR = 0.24, 95% CI: 0.59–2.59, *p* < 0.001) were significantly correlated with osteoporosis risk. In other words, respondents with poor physical fitness were 2.18 times as likely to have osteoporosis as those with good physical fitness, and those exposed to sunlight for 30 min or longer every day were 0.24 times as likely to have osteoporosis as those exposed to sunlight for less than 30 min every day. Accordingly, institutions must encourage employees to spend more time in the sun every day and improve their physical fitness through exercise.

## 1. Introduction

Osteoporosis-induced bone fracture is a serious public health problem that can result in serious diseases or early death [1]. Osteoporosis is a silent disease: it is difficult to notice at an early clinical stage, and only after the occurrence of spinal or hip fracture can a severe loss in bone density be detected [2]. Factors leading to bone fracture are classified into direct (congenital) and indirect (acquired) factors. Direct factors include age, body type, heredity, hormones, sex, and ethnicity. People with slimmer bodies or who are older are more vulnerable to osteoporosis. Hereditary factors, such as family medical history, are also critical determinants of osteoporosis risk. Generally, women are 6–8 times as vulnerable to osteoporosis as men are; for women, bone density declines rapidly after menopause or decreased gonad function. Indirect factors primarily comprise environmental factors and health habits, such as smoking, excessive alcohol consumption, excessive caffeine consumption, lack of exercise, insufficient exposure to sunlight, insufficient calcium intake, insufficient or excessive protein intake, and long-term medication use [2,3,4,5,6].

A study in Singapore reported that only 58% of women aged 45 years or older had learned about osteoporosis, and that 69.5% were unaware that family medical history constitutes a critical risk factor [7]. A study in Taiwan reported that 15% of female urban residents were unaware of the threat of osteoporosis, and 15.5% did not consider it a serious disease [8]. According to an osteoporosis awareness survey of middle-aged men by Hou et al. [9], 35% of the respondents thought that osteoporosis is only concerning for women with menopause, and 40% thought that osteoporosis is irrelevant to their health; furthermore, as for osteoporosis-induced fracture, 81% did not believe that osteoporosis can lead to death, and 91% underestimated the disease’s fatality rate. Knowledge of osteoporosis must therefore be improved in the general public.

According to a survey by Chen et al. [10] of public health nurses responsible for public health education, 49.0% of respondents falsely considered aging-related declines in body height to be normal; 46.8% falsely considered osteoporosis to be easy to treat and diagnose, with no special attention required; and only 47.5% correctly identified the tools for diagnosing the disease. Because osteoporosis occurs silently and slowly, it is difficult to detect and treat at its early stage if medical personnel do not take the initiative to inquire about and examine the disease. In an investigation by Chang et al. [11] on how much doctors knew about osteoporosis prevention, although 88.8% of the respondents had diagnosed osteoporosis in patients, only 53.3% had continued to diagnose new cases of the disease every week. This indicates an insufficient awareness of osteoporosis among these doctors. Furthermore, only 51.3% of the respondents would actively discuss osteoporosis-related topics with others. Therefore, knowledge of osteoporosis must be improved among medical personnel. 

In particular, caregivers in institutional workplaces are particularly vulnerable to bone and muscle diseases over the long term. This is because of their hermetic work environment, insufficient exposure to sunlight, and laborious work in taking care of patients. Moreover, most of these caregivers are at middle age or older, making them highly vulnerable to osteoporosis. Therefore, using the One-Minute Osteoporosis Risk Test, this study investigated risk factors for osteoporosis for caregivers in institutions caring for persons with disabilities.

## 2. Method

This cross-sectional study analyzed data from the Statistics on Welfare Institutions and Workers for the Disabled in 2015 covering the 271 disability institutions in Taiwan, which together employ 9449 caregivers [12]. This study excluded institutions in Fujian Province (offshore islands), analyzing data for the remaining 9400 caregivers as the study population. The caregivers include administrators, social workers, nursing personnel, instructors, life attendants, and trainers. According to the sample size calculator by Raosof, Inc. [13] (for a 95% confidence interval [CI] and 5% sampling error), a representative sample must have at least 370 people.

Data on osteoporosis risk factors and osteoporosis knowledge were collected for analysis through a structured questionnaire, which passed reliability and validity tests. Seven disability welfare institutions mainly caring for people with intellectual disabilities or related multiple disabilities, were selected as research sites. Of the 465 copies of the questionnaire distributed to the caregivers, 455 valid responses were returned for a valid return rate of 98%.

Specifically, the questionnaire asked caregivers about study informed consent firstly, and then their demographic data, health habits, and working style. Risk of osteoporosis was measured using the Chinese edition of the One-Minute Osteoporosis Risk Test. This Chinese version was translated and edited by the Taiwanese Osteoporosis Association, according to the original version provided by the International Osteoporosis Foundation. The risk test evaluates several crucial risk factors in 19 items, which together have a total score of 19. A higher score indicates having more osteoporosis risk factors, and those with a high score must visit a specialist or have their bone density measured in a hospital. Specifically, according to research conducted outside of Taiwan, a score of 5 and 6.5 indicate a 70% and 100% risk of having osteoporosis, respectively; the osteoporosis risk test thus aids the detection of early-stage osteoporosis [14].

Questionnaire responses were coded in Microsoft Excel 2010 and analyzed in SPSS for Windows (Version 20.0). Descriptive-statistical frequency, a χ^2^ test, and logistic regression analysis were used to analyze the correlation between demographic variables, health habits, working style, and osteoporosis risk.

## 3. Results

The demographic characteristics of the respondents revealed that female caregivers accounted for 83.7%, and male for 16.3%. The average age of respondents was 43.5 ± 11.5 years old (range = 20–76 years), with more than fifty percent possessing college and higher degrees. The respondents reported that 32.5% had chronic disease diagnosis and 22.9% accepted long-term medication currently. With regard to BMI among the respondents, 32.5% were obese (BMI: ≥27), 28.9% were overweight (BMI: 24–26.9), 47.1% were normal (BMI: 18.5–23.9) and 3.5% were underweight (BMI: <18.5). 

Table 1 lists respondent data, segmented by osteoporosis risk; 395 (86.8%) and 60 (13.2%) of respondents scored <5 and ≥5, respectively. Table 2 and Table 3 present the distribution for nonmodifiable osteoporosis risk factors. As noted in Table 3, the primary lifestyle-associated risk factors were exercising for less than 30 min daily (38%), insufficient dairy product or calcium tablet intake (28%), and engaging in outdoor activities for less than 10 min daily or not taking vitamin D supplements (29.9%).

In the univariate analysis between demographic data and osteoporosis risk, age (*p* < 0.002), education level (*p* < 0.001), disease diagnosis (*p* < 0.001), and long-term medication (*p* < 0.001) were significantly correlated with osteoporosis risks. This means that people who are prone to experiencing osteoporosis are aged 50 years or older, have low education levels, have diagnoses of other diseases, and have undergone long-term medication. Sex and body mass index were not significantly correlated with osteoporosis risk (Table 4). As for health habits, osteoporosis risk was significantly and positively correlated with having undergone bone density tests (*p* = 0.013) as well as physical fitness (*p* < 0.001), dietary habits (*p* = 0.048), and average time of exposure to sunlight (*p* < 0.001). Regular exercise and an individual’s health condition were not significantly correlated with osteoporosis risk (Table 5). As for working style, osteoporosis risk was not significantly correlated with job title (*p* = 0.219), number of days at work (*p* = 0.632), number of hours at work (*p* = 0.395), shift work (*p* = 0.066), and work pattern (*p* = 0.196), as detailed in Table 6.

After the univariate analysis, logistic regression analysis was then conducted for the eight variables that were significantly correlated with osteoporosis risks, which were age, education level, prior disease diagnosis, having undergone bone density tests, long-term medication use, physical fitness, dietary habits, and average daily time of exposure to sunlight. The results are presented in Table 7. Model 1 tested whether the demographic factors predict osteoporosis risks, and its regression results revealed that respondents aged 50 years or older (odds ratio [OR] = 5.52, 95% CI = 1.17–25.93, *p* = 0.03) were 5.52 times as likely to experience osteoporosis as those aged 18–29 years. After physical fitness, dietary habits, and average daily time of exposure to sunlight were added into Model 2, age exhibited no significant correlation with osteoporosis risks. The results for Model 2 also indicated that physical fitness (OR = 2.18, 95% CI = 1.12–4.27, *p* = 0.023) and average time of exposure to sunlight (OR = 0.24, 95% CI = 0.59–2.59, *p* < 0.001) were significantly correlated with osteoporosis risk. This means that respondents with poor physical fitness were 2.18 times as likely to experience osteoporosis as those with good physical fitness, and that those who have an average daily time of exposure to sunlight for 30 min or longer were 0.24 times as likely to experience osteoporosis as those who had an average of less than 30 min of sunlight exposure every day. 

## 4. Discussion

Osteoporosis screening and risk factor evaluation allow clinicians to determine which groups require follow-up interventions that reduce their risks of disease and death [15]. According to research conducted outside Taiwan, people who score 5 points or higher in the One-Minute Osteoporosis Risk Test have a 70% risk of getting osteoporosis; this test is thus a reference for clinicians wishing to identify the risk of osteoporosis early. In the present study, 86.8% of the respondents scored less than 5. In another study of women in Taiwan, a score of >5 in the One-Minute Osteoporosis Risk Test indicated a 27% risk of getting osteoporosis [16]. Many other studies which used the One-Minute Osteoporosis Risk Test results varied due to the cut off points and different subjects [17,18]. In this study, for improved diagnosis, a more informative score threshold than the present one of 5 should be determined. Whether early risk factors can lead to osteoporosis requires diagnosis by specialists; clinicians must pay attention to the number of risk factors in an individual because it indicates osteoporosis risk.

The intake of calcium tablets and vitamin D supplements is regarded as an effective means of preventing and treating osteoporosis [19] as well as bone fracture for people at middle age or older [20] and women at menopause [21]. However, calcium and vitamin D supplements might help bone health at the expense of health risk, as Razzaque [22] revealed that it is likely that calcium and phosphorus dysregulation, induced by exogenous vitamin D supplementation, may lead to tissue and organ damages, even without developing hypervitaminosis D. 

A study of ethnic Chinese people indicated that family bone fracture history, decline in body height, and premature menopause increase the osteoporosis risk [23]. Independent factors include age, bone fracture history or family medical history, premature menopause, and long-term medication use [24]. Awareness raising with regard to osteoporosis risk factors is most crucial to preventing and treating the disease.

Osteoporosis is a serious risk factor for bone fracture [25], which has a large medical cost [26,27] and social burden [28]. Some countries have established bone fracture reporting systems to mitigate these costs [29]. Taiwan has also established a medical network for preventing and treating osteoporosis-induced bone fracture [30]. However, a study in Australia indicated that osteoporosis diagnosis and treatment in primary care remained insufficient and must be addressed [31]. Medical education, encouraging a habit of exercise, and fall prevention help prevent osteoporosis [32].

This study noted that 13.2% of institutional caregivers are vulnerable to osteoporosis. Primary risk factors include insufficient daily exercise and insufficient outdoor activities as well as a lack of dairy products, calcium tablets, and vitamin D supplement intake. Health management in the workplace requires promoting lifestyle adjustments, such as a healthier diet and engaging in healthy activities. According to this study’s logistic regression results, insufficient exercise and insufficient exposure to sunlight greatly increase employees’ risk of early-stage osteoporosis. Since hypovitaminosis D status usually reflects reduced sunlight exposure, the obvious primary replacement should be safe sunlight exposure, and not only dependency on supplements, thus avoiding related adverse effects [33]. Because of its cross-sectional design, this study could not demonstrate a causal relationship. Moreover, because the sources of samples are difficult to identify, random sampling could not be conducted. Therefore, convenience sampling was used instead on our target population of caregivers working in disability institutions.

## 5. Conclusions

This study analyzed the osteoporosis risk factors in caregivers in disability welfare institutions, where 13.2% of the respondents were vulnerable to early-stage osteoporosis. According to an independent variable analysis, the primary osteoporosis risks factors were age, education level, having undergone bone density tests, prior disease diagnosis, long-term medication use, physical fitness, dietary habits, and average daily time of exposure to sunlight. Multivariate analysis revealed that physical fitness and average daily time of exposure to sunlight were significantly correlated with osteoporosis risks. The present study is one of the first to estimate osteoporosis risk on institutional caregivers based on the IOF One-Minute Osteoporosis Risk Check, and there is no comparable study yet. However, to improve bone health for caregivers, the managers at disability welfare institutions must pay attention to the risk factors when formulating health promotion plans.

## Figures and Tables

**Table 1:** Osteoporosis risk groups *.

Risk Groups **	n	%
<5	395	86.8
≧5	60	13.2

* Range: 0–16 for men; 0–18 for women. ** A score of ≥ 5 in the One-Minute Osteoporosis Risk Test indicates a high risk of osteoporosis; a “yes” response to an item does not mean that the respondent has osteoporosis but that the respondent exhibits the corresponding risk factor, and thus has a greater risk of osteoporosis.

**Table 2:** **International Foundation for Osteoporosis** (IOF One-Minute Osteoporosis Risk Test of caregivers (n = 455).

Non-Modifiable Risk Factors *	n	%
1. Have either of your parents been diagnosed with osteoporosis or broken a bone after a minor fall (a fall from standing height or less)?		
Yes	58	12.7
No	397	87.3
2. Did either of your parents have a stooped back (dowager’s hump)?		
Yes	50	11.0
No	405	89.0
3. Are you 40 years old or older?		
Yes	270	59.3
No	185	40.7
4. Have you ever broken a bone after a minor fall, as an adult?		
Yes	38	8.4
No	417	91.6
5. Do you fall frequently (more than once in the last year) or do you have a fear of falling because you are frail?		
Yes	25	5.5
No	430	94.5
6. After the age of 40, have you lost more than 3 cm in height (just over 1 inch)?		
Yes	44	90.3
No	411	9.7
7. Are you underweight (is your Body Mass Index less than 19 kg/m^2^)?		
Yes	22	4.8
No	433	95.2
8. Have you ever taken corticosteroid tablets (cortisone, prednisone, etc.) for more than three consecutive months?		
Yes	21	4.6
No	434	95.4
9. Have you ever been diagnosed with rheumatoid arthritis?		
Yes	19	4.2
No	436	95.8
10. Have you been diagnosed with an over-active thyroid, overactive parathyroid glands, type 1 diabetes or a nutritional/gastrointestinal disorder such as Crohn’s or celiac disease?		
Yes	19	4.2
No	436	95.8

* A score of ≥5 in the One-Minute Osteoporosis Risk Test indicates a high risk of osteoporosis; a “yes” response to an item does not mean that the respondent has osteoporosis but that the respondent exhibits the corresponding risk factor, and thus has a greater risk of osteoporosis.

**Table 3:** IOF One-Minute Osteoporosis Risk Test of caregivers (non-modifiable and lifestyle risk factors) (n = 455).

Non-Modifiable Risk Factors (cont.)	n	%
For Women:		
11. For women over 45: Did your menopause occur before the age of 45?		
Yes	40	10.5
No	341	89.5
12. Have your periods ever stopped for twelve consecutive months or more (other than because of pregnancy, menopause or hysterectomy)?		
Yes	25	6.6
No	356	93.4
13. Were your ovaries removed before age 50, without you taking Hormone Replacement Therapy?		
Yes	11	2.9
No	370	97.1
For Men:		
14. Have you ever suffered from impotence, lack of libido or other symptoms related to low testosterone levels?		
Yes	3	4.1
No	71	95.9
**Lifestyle risk factors**	**n**	**%**
15. Do you regularly drink alcohol in excess of safe drinking limits (more than two units a day)?		
Yes	11	2.4
No	444	97.6
16. Do you currently, or have you ever, smoked cigarettes?		
Yes	40	8.8
No	415	91.2
17. Is your daily level of physical activity less than 30 min per day (housework, gardening, walking, running etc.)?		
Yes	173	38.0
No	282	62.0
18. Do you avoid, or are you allergic to milk or dairy products, without taking any calcium supplements?		
Yes	131	28.8
No	324	71.2
19. Do you spend less than ten minutes per day outdoors (with part of your body exposed to sunlight), without taking vitamin D supplements?		
Yes	136	29.9
No	319	70.1

**Table 4:** Univariate analysis of demographic data and osteoporosis risk (n = 455).

	<5	≧5		
Variable	n (%)	n (%)	χ2	*p*-Value
Sex			0.081	0.776
M	65 (87.8)	9 (12.2)		
F	330 (86.6)	51 (13.4)		
Age			46.394	<0.001
18–29	68 (97.1)	2 (2.9)		
30–39	108 (98.2)	2 (1.8)		
40–49	112 (88.2)	15 (11.8)		
≥50	107 (72.3)	41 (27.7)		
BMI * (n = 454)			1.352	0.717
Underweight	15 (93.8)	1 (6.3)		
Normal	188 (87.9)	26 (28.3)		
Overweight	112 (85.5)	19 (14.5)		
Obese	79 (84.9)	14 (15.1)		
Education level			35.782	<0.001
Elementary school	16 (61.5)	10 (38.5)		
Junior high school	26 (81.3)	6 (18.8)		
Senior high school	128 (80.0)	32 (20.0)		
College	214 (94.7)	12 (5.3)		
Graduate school or above	11 (100.0)	0 (0.0)		
Disease diagnosis			15.90.3	<0.001
No	280 (91.2)	27 (8.8)		
Yes	115 (77.7)	33 (22.3)		
Long-term medication			13.868	<0.001
No	316 (90.0)	35 (10.0)		
Yes	79 (76.0)	25 (24.0)		

**Table 5:** Univariate analysis of healthy lifestyle and osteoporosis risk (n = 455).

	<5	≥5		
Variable	n (%)	n (%)	χ2	*p*-Value
Health condition			5.554	0.235
Very healthy	38 (90.5)	4 (9.5)		
Healthy	159 (86.9)	24 (13.1)		
Moderate	169 (86.2)	27 (13.8)		
Unhealthy	27 (90.0)	3 (10.0)		
Very unhealthy	2 (50.0)	2 (50.0)		
Bone density test			6.127	0.013
Yes	216 (83.4)	43 (16.6)		
No	179 (91.3)	17 (8.7)		
Physical fitness			16.043	<0.001
Satisfactory	234 (92.5)	19 (7.5)		
Unsatisfactory	161 (79.7)	41 (20.3)		
Dietary habits			7.918	0.048
Very poor	8 (100.0)	0 (0.0)		
Poor	120 (82.2)	26 (17.8)		
Moderate	242 (87.7)	34 (12.3)		
Satisfactory	25 (100.0)	0 (0.0)		
Exercise			0.073	0.786
No	112 (87.5)	16 (12.5)		
Yes	283 (86.5)	44 (13.5)		
Average daily time of exposure to sunlight			33.350	<0.001
<30 min	119 (74.4)	41 (25.6)		
≥30 min	276 (93.6)	19 (6.4)		

**Table 6:** Univariate analysis of working style and osteoporosis risk (n = 455).

	<5	≥5		
Variable	n (%)	n (%)	χ2	*p*-Value
Job title			1.508	0.219
First-line	272 (85.5)	46 (14.5)		
Non-first-line	123 (89.8)	14 (10.2)		
Days at work weekly			0.229	0.632
≤5 days	269 (87.3)	39 (12.7)		
>5 days	126 (85.7)	21 (14.3)		
Hours at work daily			0.724	0.395
≤8 h	296 (86.0)	48 (14.0)		
>8 h	99 (89.2)	12 (10.8)		
Shift work			3.372	0.066
Yes	161 (83.4)	32 (16.6)		
No	234 (89.3)	28 (10.7)		
Work pattern			6.039	0.196
Primarily static	26 (78.8)	7 (21.2)		
Mostly static	56 (93.3)	4 (6.7)		
Half static, half mobile	166 (87.4)	24 (12.6)		
Mostly mobile	106 (87.6)	15 (12.4)		
Primarily mobile	41 (80.4)	10 (19.6)		

**Table 7:** Logistic regression analysis of osteoporosis risk factors (n = 455).

	Model 1		Model 2	
Variable	OR (95% C.I)	*p*-Value	OR (95% C.I)	*p*-Value
Constant	0.002		0.000	
Age (30–39 vs. 18–29)	0.465 (0.06–3.46)	0.46	0.39 (0.48–3.10)	0.37
Age (40–49 vs. 18–29)	2.58 (0.54–12.41)	0.24	2.28 (0.43–12.20)	0.33
Age (≥50 vs. 18–29)	5.52 (1.17–25.93)	0.03	5.31 (0.99–28.46)	0.051
Education level (junior high school vs. elementary school)	0.57 (0.16–1.95)	0.37	0.78 (0.20–3.06)	0.72
Education level (senior high school vs. elementary school)	0.75 (0.30–1.91)	0.55	1.11 (0.39–3.18)	0.85
Education level (college vs. elementary school)	0.29 (0.10–0.82)	0.20	0.52 (0.16–1.70)	0.28
Education level (graduate school or above vs. elementary school)	0.00 (0.00)	0.99	0.00 (0.00)	0.99
Disease diagnosis (No)	1.52 (0.74–3.11)	0.26	1.48 (0.70–3.11)	0.30
Long-term medication (No)	1.24 (0.59–2.59)	0.58	1.41 (0.64–3.07)	0.39
Bone density test (No)			1.31 (0.64–2.68)	0.45
Physical fitness (Satisfactory)			2.18 (1.12–4.27)	0.023
Average daily time of exposure to sunlight (<30 min)			0.24 (0.59–2.59)	<0.001
